# Cytotoxicity, mutagenicity and genotoxicity of electronic cigarettes emission aerosols compared to cigarette smoke: the REPLICA project

**DOI:** 10.1038/s41598-023-44626-1

**Published:** 2023-10-30

**Authors:** Rosalia Emma, Virginia Fuochi, Alfio Distefano, Konstantinos Partsinevelos, Sonja Rust, Fahad Zadjali, Mohammed Al Tobi, Razan Zadjali, Zaina Alharthi, Roberta Pulvirenti, Pio Maria Furneri, Riccardo Polosa, Ang Sun, Massimo Caruso, Giovanni Li Volti, the Replica Project Group, Giovanni Li Volti, Massimo Caruso, Rosalia Emma, Antonio Giordano, Ang Sun, Vladislav Volarevic, Ronny Lesmana, Konstantinos Poulas, Alfio Distefano, Konstantinos Partsinevelos, Roberta Pulvirenti, Aurora Costa, Aleksandar Arsenijevic, Melisa I. Barliana, Konstantinos Mesiakaris, Najwa Albalushi, Chiara Giardina, Salvatore Furnari

**Affiliations:** 1 Department of Clinical and Experimental Medicine, University of Catania, Via S. Sofia, 97, 95123 Catania, Italy; 2 Center of Excellence for the Acceleration of Harm Reduction (CoEHAR), University of Catania, Via S. Sofia, 97, 95123 Catania, Italy; 3 Department of Biomedical and Biotechnological Sciences, University of Catania, Via S. Sofia, 97, 95123 Catania, Italy; 4 ECLAT Srl, Spin Off of the University of Catania, Via. S Sofia 89, 95123 Catania, Italy; 5 Department of Clinical Biochemistry, College of Medicine and Health Sciences, Sultan Qaboos University, P.C 123, P.O. Box 35, Khodh, Oman; 6 Department of Biology, College of Science and Technology, Sbarro Institute for Cancer Research and Molecular Medicine, Temple University, Philadelphia, USA; 7 Faculty of Medical Sciences, Center for Molecular Medicine and Stem Cell Research, University of Kragujevac, Kragujevac, Serbia; 8 Center of Excellence for Pharmaceutical Care Innovation, Universitas Padjadjaran, Jl. Raya Bandung Sumedang KM. 21, Jatinangor, 45363 Indonesia; 9 Department Biomedical Sciences, Faculty of Medicine, Universitas Padjadjaran, Jl. Raya Bandung Sumedang KM. 21, Jatinangor, 45363 Indonesia; 10 Institute for Research and Innovation, IRIS, Patras Science Park, Patras, Greece; 11 Laboratory of Molecular Biology and Immunology, Department of Pharmacy, University of Patras, Patras, Greece; 12 Department of Biological Pharmacy, Biotechnology Laboratory, Faculty of Pharmacy, Universitas Padjadjaran, Jl. Raya Bandung Sumedang KM. 21, Jatinangor, 45363 Indonesia

**Keywords:** Pathogenesis, Biological models

## Abstract

Concerns have recently increased that the integrity of some scientific research is questionable due to the inability to reproduce the claimed results of some experiments and thereby confirm that the original researcher's conclusions were justified. This phenomenon has been described as 'reproducibility crisis' and affects various fields from medicine to basic applied sciences. In this context, the REPLICA project aims to replicate previously conducted in vitro studies on the toxicity of cigarette smoke and e-cigarette aerosol, sometimes adding experiments or conditions where necessary, in order to verify the robustness and replicability of the data. In this work the REPLICA Team replicated biological and toxicological assessment published by Rudd and colleagues in 2020. As in the original paper, we performed Neutral Red Uptake (NRU) assay for the evaluation of cytotoxicity, Ames test for the evaluation of mutagenesis and In Vitro Micronuclei (IVMN) assay for the evaluation of genotoxicity on cells treated with cigarette smoke or e-cigarette aerosol. The results showed high cytotoxicity, mutagenicity and genotoxicity induced by cigarette smoke, but slight or no cytotoxic, mutagenic and genotoxic effects induced by the e-cigarette aerosol. Although the two studies presented some methodological differences, the findings supported those previously presented by Rudd and colleagues.

## Introduction

In recent years, there has been a growing interest in electronic cigarettes (e-cigarettes) as a potentially safer alternative to traditional tobacco products. A safety review conducted by the Committee on Toxicity of Chemicals in Food, Consumer Products, and the Environment (COT) concluded that when e-cigarettes are manufactured and used correctly, the risk of adverse health effects is significantly lower compared to combustible tobacco cigarettes. However, there remains some uncertainty regarding the potential health risks associated with inhaling flavorings and thermally-derived products from e-cigarettes^1^.

The concern for public health and regulatory policies surrounding the toxicological aspects of vapor products has gained global attention. This was primarily triggered by reported cases of lung injury (referred to as EVALI) associated with improper use of e-cigarettes for tetrahydrocannabinol (THC) consumption and the use of vitamin E additives^2^. Several countries, including India, Australia, Oman, Egypt, Colombia, among others, have banned e-cigarettes, while others have implemented regulations for the marketing of e-cigarettes and e-liquids. Regulatory bodies such as the U.S. Food and Drug Administration (FDA) and the European Union (EU) have issued requirements and guidance to regulate premarket tobacco product applications for electronic nicotine delivery systems^3,4^.

To assess the toxicological potential of e-cigarettes, international guidelines, such as the International Conference on Harmonisation S2(R1) (2011), the UK Committee on Mutagenicity of Chemicals in Food, Consumer Products, and the Environment (2011), Health Canada (2005), and the Cooperation Centre for Scientific Research Relative to Tobacco (CORESTA) (2004), recommend the use of a battery of in vitro tests as part of the pre-clinical assessment strategy. These guidelines call for the evaluation of various toxicological endpoints using multiple assays, including the bacterial reverse mutation (Ames) assay for mutagenicity, in vitro micronucleus (IVMN) assay for genotoxicity, and the neutral red uptake (NRU) assay for acute cytotoxicity evaluation^5–7^. These three in vitro toxicity tests are commonly used as standard assays to assess the toxicity of tobacco products and e-cigarettes^8^.

Major e-cigarette manufacturers have published studies evaluating their products, including emissions, cytotoxicity, genotoxicity, and mutagenicity data^9–12^. Independent replication of these studies is crucial to verify the findings and establish the credibility of the data, supporting the regulation of electronic cigarettes. Incorrect or flawed results can misinform policies and have detrimental effects on research practices, eroding public trust in science and, ultimately, impacting health and social care practices. To address this issue, the multicenter REPLICA project was initiated to replicate high-profile studies conducted by tobacco companies' research and development (R&D) departments, aiming to assess the validity of the original work under scrutiny (https://replica.coehar.org/).

During the final phase of the REPLICA project, the Italian team (CoEHAR, University of Catania-LAB-A) and their partner in Oman (Sultan Qaboos University-LAB-B) conducted a replication study of a paper published by Rudd and colleagues from Imperial Brands PLC^9^. This paper provides a summary of comparative data on aerosol emissions and in vitro toxicity, utilizing the neutral red uptake (NRU), bacterial reverse mutation (Ames), and in vitro micronucleus (IVMN) assays. The study focused on a pod-system e-cigarette (myblu; Imperial Brands PLC, Bristol, United Kingdom) in comparison to 3R4F reference combustible cigarette (University of Kentucky). The researchers observed that many of the harmful and potentially harmful components present in combustible cigarette smoke were not detected in e-cigarette aerosol. Through established in vitro biological tests, the e-cigarette aerosol did not exhibit any mutagenic or genotoxic activity under the given test conditions. In contrast, the 3R4F cigarette smoke demonstrated mutagenic and genotoxic activity. Additionally, the e-cigarette aerosol was found to be 300 times less cytotoxic than combustible cigarette smoke according to the neutral red uptake assay.

In this study, we performed a replication of the in vitro biological tests to examine the cytotoxic, mutagenic, and genotoxic activity of myblu e-cigarette aerosol compared to 1R6F combustible cigarette smoke (University of Kentucky). We employed similar methods to those used by Rudd and colleagues to evaluate and validate their findings^9^.

## Results

### Cytotoxicity: effect of cigarette whole smoke and *my*blu whole aerosol on cell viability.

**Figure 1:**
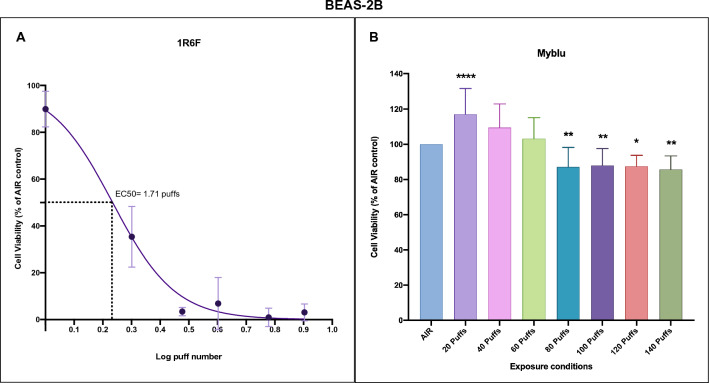
Cytotoxicity evaluation of BEAS-2B cells after exposure to 1R6F combustible cigarette smoke and myblu aerosol. (**A**) Dose response curve of BEAS-2B cells exposed to 1R6F smoke showed an EC_50_ value of 1.71 puffs (Log EC_50_ = 0.23 puff). The optical density (OD) means ± standard deviation (SD) of negative controls (AIR, INC and ALI) were 0.138 ± 0.03, 0.129 ± 0.02 and 0.117 ± 0.01, respectively. (**B**) Barplot representing the BEAS-2B cell viability results after exposure to myblu aerosol. The OD means ± SD of negative controls (AIR, INC and ALI) for *my*blu were 0.077 ± 0.02, 0.116 ± 0.04 and 0.102 ± 0.01, respectively. All data are reported as percentage of the respective AIR control and displayed as mean ± SD from 9 replicated wells from triplicate Transwell inserts of two independent experiments (LAB-A and LAB-B). *P < 0.05; **P < 0.01; ****P < 0.0001.

After exposure to whole smoke from 1R6F reference cigarettes, BEAS-2B cell viability drastically decreased as early as 2 puffs until complete cell death at 4 puffs with an EC_50_ value of 1.71 puffs (Fig. 1A). Instead, unlike Rudd and colleagues, the EC_50_ value could not be calculated for *my*blu exposure due to low cytotoxicity at 140 puffs (Fig. 1B). We observed a reduced cell viability starting from 80 to 140 puffs, which did not decrease below the 80% of viability. Particularly, significant decrease of cell viability was observed for 80 (*P* = 0.003), 100 (*P* = 0.008), 120 (*P* = 0.038) and 140 puffs (*P* = 0.004) compared to the AIR control.

In addition to Rudd and colleagues, we observed microscopically the cells exposed to both 1R6F smoke and *my*blu aerosol at 24, 48, and 65 h. Exposure of BEAS-2B cells to 1R6F smoke induced morphological changes of cells with alterations in cell volume, nucleus volume, and cell sphericity at all the time-points (Fig. S1 of supplementary material). Similar morphological changes of BEAS-2B cells were observed after exposure to *my*blu aerosol starting from 80 puffs at 24 h. But a reversal of morphological changes was observed from 48 h to complete recovery at 65 h (Fig. S2 of supplementary material).

### Mutagenicity effect of 1R6F cigarette smoke and *my*blu aerosol

The negative controls (Solvent control) were in the normal range based on our laboratory experience and to literature data^3^. Also, the positive controls (i.e., Chem Controls; Sodium Azide, Daunomicyn, and 2-Aminoanthracene) were in the range reported in the manufacturing manual (Trinova Biochem GmbH-Germany). No significant differences were observed between Solvent controls and AIR controls for all the tested conditions. Instead, significant differences were observed between Solvent controls or AIR controls and the respective Chem Controls (*P* < 0.0001).

**Figure 2:**
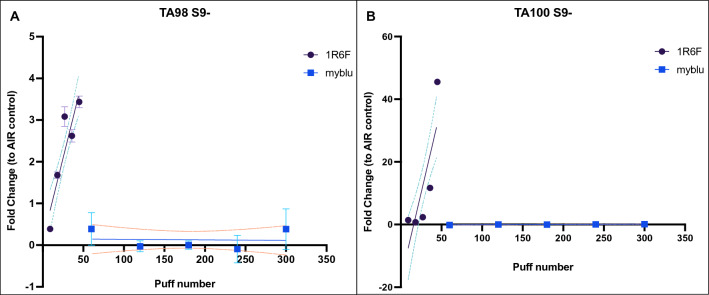
Mutagenicity evaluation by Ames test in *Salmonella typhimurium* TA98 (**A**) and TA100 (**B**) without S9 metabolic activation after exposure to 1R6F combustible cigarette smoke (violet round dot) or myblu aerosol (blue square dot). The means ± standard deviation (SD) of revertants (N) in solvent controls for TA98 and TA100 of 1R6F experiments were 39.46 ± 35.7 and 104 ± 67.7, respectively. The means ± standard deviation (SD) of revertants (N) in solvent controls for TA98 and TA100 of *my*blu experiments were 9.5 ± 2.7 and 85.17 ± 13.3, respectively. Data are reported as Fold change to AIR control. Each data point represents the mean ± SD from triplicate Petri dishes. The dashed lines represent the 95% confidence interval of the regression line.

Exposure to 1R6F combustible cigarette smoke induced significant increase in revertants in a dose-dependent manner without S9 metabolic activation. Indeed, significant dose-dependent increase in revertants was observed in both TA98 (up to approximately 3.5-fold change to AIR control; *P* < 0.0001) and TA100 (up to 45-fold change to AIR control; *P* < 0.0001) without S9 metabolic activation after exposure to 1R6F cigarette smoke. The exposure to *my*blu aerosol did not induce any significant increase in revertants in both strains (Fig. 2). The linear regression results of mutagenic activity in *Salmonella typhimurium* (TA98 and TA100) without S9 metabolic activation are reported in Table S2 (supplementary material).

**Figure 3:**
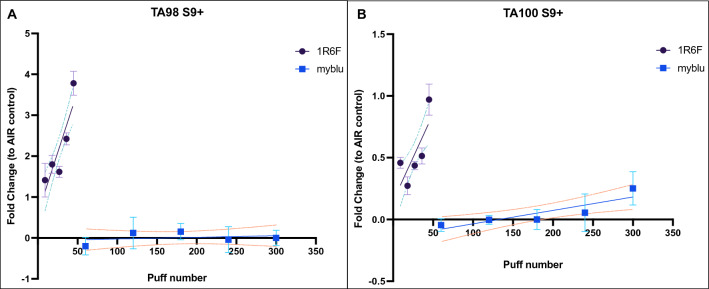
Mutagenicity evaluation by Ames test in *Salmonella typhimurium* TA98 (**A**) and TA100 (**B**) with S9 metabolic activation after exposure to 1R6F combustible cigarette smoke (violet round dot) or myblu aerosol (blue square dot). Data are reported as Fold change to AIR control. The means ± standard deviation (SD) of revertants (N) in solvent controls for TA98 and TA100 of 1R6F experiments were 32.08 ± 13.7 and 79.5 ± 16.5, respectively. The means ± SD of revertants (N) in solvent controls for TA98 and TA100 of *my*blu experiments were 11.58 ± 3.9 and 79.75 ± 10.5, respectively. Each data point represents the mean ± SD from triplicate Petri dishes. The dashed lines represent the 95% confidence interval of the regression line.

Similar results were observed when the Ames assay was performed for both TA98 (up to nearly fourfold change to AIR control; *P* < 0.0001) and TA100 (up to onefold change to AIR control; *P* = 0.002) with S9 metabolic activation. No significant increase in revertants (linear slope did not differ from zero; *P* = 0.91) was observed for TA98 strain with S9 metabolic activation after exposure to *my*blu aerosol. Instead, a slight increase of revertants for TA100 S9 + (up to approximately 0.2-fold change to AIR control) was observed after *my*blu aerosol exposure, with a linear slope significantly different from zero (*P* = 0.005) (Fig. 3). The linear regression results of mutagenic activity in *Salmonella typhimurium* (TA98 and TA100) with S9 metabolic activation are reported in Table S1 (supplementary material).

### Genotoxic effect of 1R6F cigarette smoke and myblu aerosol

Due to different exposure system, not able to perform smoke dilution, we performed a dose–response curve in order to establish the EC_50_ dose for the V79 cells exposed to 1R6F combustible cigarette whole smoke: the calculated EC_50_ value for V79 cells was 3.149 puffs (Fig. S3 of supplementary material). We then performed the IVMN assay with and without S9 metabolic activation after exposure from 1 to 4 puffs of 1R6F combustible cigarette smoke. For *my*blu exposure the same puff numbers by Rudd et al. (2020) were used (20–100 puffs).

No difference was shown among the three negative controls, cells left to grow in the incubator submerged in the culture medium (INC), cells left to grow in the incubator with the apical side exposed to the air in air–liquid interface mode (ALI) and cells exposed to puffs of particulate-filtered laboratory air using a Cambridge Filter Pad (AIR) for both IVMN with and without S9 activation. Instead, the positive controls, including cyclophosphamide A (for IVMN S9 +) and mitomycin C (for IVMN S9-), were significantly increased compared to the respective AIR controls (Cyclo A *P* < 0.0001; Mito C *P* = 0.002).

**Figure 4:**
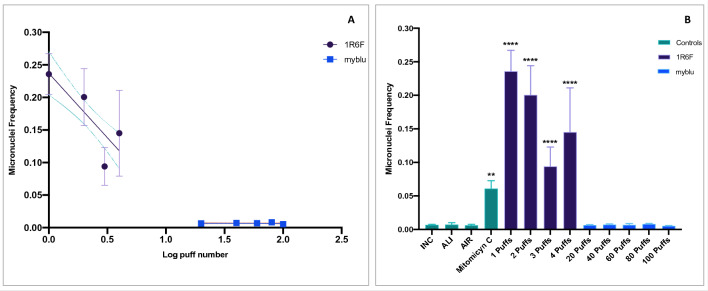
Genotoxicity evaluation by in vitro micronucleus assay without S9 activation in V79 cells after exposure to undiluted 1R6F combustible cigarette smoke or myblu aerosol at the air liquid interface. (**A**) Linear slopes of the dose–response IVMN results for 1R6F (violet round dot) and myblu (blue square dot). The dashed lines represent the 95% confidence interval of the regression line. (**B**) Barplot representing IVMN results, including both negative (INC; ALI; AIR) and positive (mitomycin C) controls, 1R6F, and myblu results. All data are reported as micronuclei frequency. Each data point or bar represents the mean ± standard deviation (SD) from 9 replicated wells from triplicate Transwell inserts. The micronuclei frequency means ± SD of negative controls (INC, ALI and AIR) were 0.007 ± 0.001, 0.007 ± 0.003 and 0.006 ± 0.002, respectively. **P < 0.01; ****P < 0.0001 compared to AIR control.

**Figure 5:**
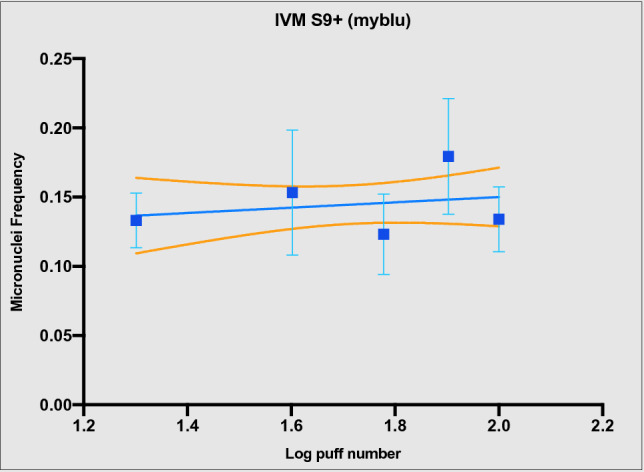
Genotoxicity evaluation by in vitro micronucleus assay with S9 activation in V79 cells after exposure to myblu aerosol (from 20 to 100 puffs) at the air liquid interface. Data are reported as micronuclei frequency. Each data point represents the mean ± standard deviation (SD) from 9 replicated wells from triplicate Transwell inserts. The dashed lines represent the 95% confidence interval of the regression line. The micronuclei frequency means ± SD of negative controls (INC, ALI and AIR) were 0.159 ± 0.03, 0.183 ± 0.05 and 0.177 ± 0.04, respectively.

The results of IVMN assay without S9 (S9-) are shown in Fig. 4. The micronuclei frequency for the 1R6F exposure, did not show a dose-dependent increase due to high cytotoxicity (Table S5 of supplementary material) of undiluted smoke (Fig. 4A). However, all the 1R6F puff numbers (from 1 to 4) induced significant increments of micronuclei frequency (*P* < 0.0001) compared to AIR control (Fig. 4B). Instead, no significant increase in micronuclei frequency was observed after exposure to *my*blu aerosol until 100 puffs (Fig. 5B and Table S6 of supplementary material).

When the IVMN assay was performed with the S9 metabolic activation, high cytotoxicity was observed for 1R6F, and less cytotoxicity was observed for *my*blu (see Tables S3 and S4 of supplementary material). Particularly, marked cytotoxicity was observed for V79 cells exposed to combustible cigarette smoke to the point of being unable to perform micronuclei counts. The cause of this cytotoxicity was attributed to the S9 mix, which is known to be cytotoxic^4^ (see Fig. S4 of supplementary material). Especially for the IVMN assay with S9 of 1R6F, the cytotoxic effect of both S9 mix and undiluted combustible cigarette smoke have added up, making the assay unfeasible. Whereas, despite the S9 cytotoxicity, we were able to perform micronuclei quantification in order to complete IVMN assay for *my*blu. The regression slope was not different from zero (*P* = 0.5) (Fig. 5), and all the micronuclei frequencies corresponding to each *my*blu puff number were not different from AIR control.

Because of the high cytotoxicity of S9 mixture and since the OECD n. 487 guideline reported that IVMN assay can be performed with or without S9 activation, we performed this assay without S9 mixture in addition to what was done by Rudd and colleagues.

## Discussion

In recent years the problem of the replicability crisis has been raised in various sectors of science. For a sensitive topic such as the regulation of products intended for human consumption, such as e-cigarettes, this issue is particularly relevant. For this reason, the Replica project set itself the objective of replicating in vitro studies that led to conclusions of significant interest, to refute or confirm their validity^5,6^. This study replicated the work by Rudd and colleagues^7^, which compared the in vitro toxicity of the *my*blu e-cigarette aerosol with that of combustible cigarette smoke. They performed a standard toxicological battery of three assays used for product assessment and regulatory applications: the NRU assay to assess cytotoxicity^8^, the bacterial reverse mutation (Ames) assay to evaluate mutagenicity^9^, and the in vitro micronucleus assay to measure genotoxicity^10^. Their results indicated that e-cigarette aerosol was low cytotoxic, and it did not show any mutagenic or genotoxic activity unlike the 3R4F cigarette smoke, which showed high cytotoxic, mutagenic and genotoxic activity.

Despite some different methodological aspects in our study, we obtained results similar to those obtained by Rudd and colleagues. The main methodological differences were: (i) the use of 1R6F reference cigarette in place of the 3R4F reference cigarette because the latter is no longer produced by the Center for Tobacco Reference Products (University of Kentucky); (ii) the different smoking and vaping apparatus. Our laboratory is equipped with separate machines, the LM1 smoking machine and the LM4E vaping machine (Borgwaldt, Hamburg, Germany). Instead, Rudd and colleagues used the SAEIVS five-port smoking/vaping machine for generation of both smoke and aerosol. Also, the SAEIVS machine is able to perform dilution of smoke/aerosol with air while the LM1 and LM4E machines were not designed to perform dilutions; (iii) the different smoke/aerosol ALI exposure in vitro system. The smoke/aerosol exposure in vitro system (SAEIVS) used by Rudd and colleagues was designed to expose cells in 96 and 24 multi-well plates, only the latter with Transwell inserts. Instead, our in vitro ALI exposure system (described in the “methods” section) allows the cell exposure with Transwell inserts of all diameters by the use of a dedicated exposure chamber. All these differences have been filled by implementing some modifications to the protocols used in the original work as described in the “methods” section.

NRU assay was performed both in LAB-A and in LAB-B. Our results confirmed the higher cytotoxicity of 1R6F cigarette smoke compared to the e-cigarette aerosol as showed by Rudd and colleagues. However, the calculated EC_50_ for the 1R6F smoke (1.71 puffs) was different from that obtained in the original work (0.236 puffs). In addition, we did not observe the same cytotoxic effect for the *my*blu aerosol. Indeed, the low cytotoxicity induced by myblu aerosol did not allow us to calculate the value of EC_50_. But we observed only a reduced cell viability, around the 80% of viability, starting from the 80 puffs to 140 puffs. These differences in results may be ascribed to the different ALI exposure apparatus. Rudd and colleagues exposed BEAS-2B cells seeded in the 96-well plate, but the cells do not have medium in the basal face of cells by this type of exposure. Then it is not a real ALI exposure because the cells are dry as the apical medium is taken to perform an air-interface exposure. As a result, part of the cytotoxicity observed by Rudd and colleagues could be due to conditions that are not optimal for normal cell health. Conversely, we exposed BEAS-2B cells using Transwell inserts placed into the exposure chamber filled with culture medium at the basal compartment that provides nutrition for cells through the Transwell membrane. This exposure apparatus provides the optimal environment to avoid the cells to dry out, especially when performing long exposures (10 to 77 min, in this case). The same ALI-exposure system combined with cytotoxicity evaluation was successfully used in our previous works^11–14^, and other published works^15,16^. Moreover, several in vitro toxicity studies used the ALI exposure with cell cultures by using appropriate apparatus developed by dedicated manufactures, such as the VITROCELL and CULTEX system, that simulate real in vivo exposure conditions^17,18^. Furthermore, we also added a morphological evaluation of cells during the recovery period at 24, 48 and 65 h (Figs. S1 and S2 in supplementary material). Thise set of experiments showed a good recovery of cells over time even though we observed some discrepancies with NRU assay results when compared to the morphological data. However, our previous study showed that NRU may present some limitation in detecting apoptotic cells^14^ especially during the early phase of this process.

The Ames test was performed only in the LAB-A, as reported by Rudd and colleagues, with *Salmonella typhimurium* TA98 and TA100 strains, which are particularly relevant for tobacco products since they have been shown to be sensitive to combustion products^19,20^. Unlike the original work, we conducted the Ames test with and without S9 metabolic activation. Our results showed that neither TA98 nor TA100 with and without S9 showed a mutagenic response after *my*blu aerosol exposure even at high doses (from 60 to 300 puffs), as opposed to what has been observed for 1R6F cigarette smoke with minor doses (from 9 to 45 puffs). These results are aligned with what Rudd and colleagues showed in their work, even though we used different exposure machines. The mutagenicity evaluation of e-cigarette aerosol by Ames assay has been also reported in literature with similar results to those reported in this work^21–24^.

Genotoxicity evaluation was conducted by LAB-A in a similar way to those reported by Rudd and colleagues, with the following exceptions: (i) we added the genotoxicity evaluation without S9 metabolic activation to improve their results; (ii) the exposure of 1R6F cigarette smoke was performed undiluted. Though, we experienced some methodological issues following their protocol. Indeed, they reported the use of S9 mix at 10%, but using the same concentration we observed a massive cell mortality (more than 50%) especially for 1R6F cigarette smoke that affects the observation of micronuclei in V79 cells. Instead, we were able to perform the micronuclei evaluation with S9 for the V79 cells exposed to *my*blu aerosol, showing no genotoxic effect. Based on literature data, we found that the S9 mix has a full set of liver metabolic enzymes, but it displays high cytotoxicity in cell-based assays^4^. Consequently, we performed a dose–response curve with different concentrations of S9 mix (from 1 to 5%), and we observed that cell viability decreased with the increment of S9 enzymatic mix percentage (Fig. S4). Probably, the high cytotoxicity levels observed in the IVMN assay with S9 are due to the sum of undiluted whole smoke cytotoxicity in addition to the S9 enzymatic mix cytotoxicity. A limitation of IVMN assay is that higher cytotoxicity levels may induce chromosome damage as a secondary effect of cytotoxicity, then it is suggested not to exceed 50% cytotoxicity^10^. Indeed, the IVMN assay without the S9 metabolic activation allowed us the micronuclei count for both 1R6F cigarette smoke and *my*blu aerosol. A high genotoxicity (exceeding the positive control) was demonstrated for 1R6F cigarette smoke at 1 puff, although no clear dose response was observed due to the cytotoxic effect of 1R6F cigarette smoke. Therefore, the results of the genotoxicity for 1R6F smoke can not be considered reliable due to the high cytotoxicity detected. However, no genotoxicity was observed for all *my*blu exposure conditions. In line with our results there is the work by Thorne et al. (2019), which showed the highest responsivity of V79 cells to combustible cigarette^25^. Another study investigated the genotoxic activity of aerosolized e-liquids in three immortalized cell lines not included in the OECD guidelines^26^, observing that in certain conditions, and with some chemical flavors, e-cigarette liquids could be able to induce genotoxicity. Moreover, the researchers reported that at the lowest dose recommended the S9 caused considerable death with the cell lines used in the study. In another study investigating the mutagenic and genotoxic potential of different chemical flavors used in e-liquids^27^, for some of them a certain mutagenic activity was observed at high concentrations on *S. typhimurium* TA100 and TA98 strains, but not for most of them. Otherwise, the IVMN test in CHO–K1 cells evidenced a certain increase in micronuclei for a low number of chemical flavors and at high concentration, with or without S9 fraction. However, all these studies were conducted with homemade systems and without following a standardized validated exposure regime, making them difficult to be replicated by other groups. All these scientific evidence on the mutagenic and genotoxic effects of e-cigarette aerosols highlight the lack of clarity still existing in this area and the need for further studies conducted following the official guidelines and that are replicable, to verify the results.

In conclusion, our findings confirmed the results on low toxicity profile of *my*blu e-cigarette obtained by Rudd and colleagues, despite some differences in methodology. Moreover, our study covered some methodological gaps and limitations in the original work, including the non-optimal ALI exposure for the cytotoxicity evaluation and improved mutagenicity and genotoxicity results by adding experiments without S9 metabolic activation as recommended in the OECD guidelines. Overall, this replication study supports the tobacco harm reduction strategy as having the potential to substantially reduce exposure to toxic combustion agents in adult smokers. Future studies are needed to advance in vitro methods in order to evaluate the long-term effects of electronic nicotine delivery systems.

## Methods

### Test products

Unlike Rudd and colleagues, we used the 1R6F reference combustible cigarette (University of Kentucky, Center for Tobacco Reference Products, Lexington, KY, USA), since the 3R4F are no longer produced by the University of Kentucky. 1R6F and 3R4F reference combustible cigarettes are very similar and only slight differences were reported regarding smoke chemistry and in vitro assays^28^. Prior to every experimental session, the 1R6F combustible cigarette were conditioned for a minimum of 48 h at 22 ± 1 °C and 60 ± 3% relative humidity, according to ISO 3402:1999^29^.

The same electronic cigarette used by Rudd and colleagues, *my*blu (Imperial Brands PLC, Bristol, United Kingdom), was used for this replication study. The *my*blu is a "closed pod-system” e-cigarette consisting of two elements: a rechargeable battery (battery capacity, 350 mAh) and a replaceable e-liquid pod with 1.5 mL volume and a coil resistance of 1.3 Ω (cartomizer). The tobacco flavored e-liquids with 1.6% (w/w) nicotine were used for the experiments. All the *my*blu e-cigarettes and *my*blu pods were purchased from Italian retailers.

### Smoke and vapour exposure

The selected products were tested on standardized equipment simulating smoking topography: the LM1 Smoking Machine (Borgwaldt, Hamburg, Germany) (Fig. S6A) was used to smoke the 1R6F combustible cigarettes following the Health Canada Intense (HCI) regime (puff volume, duration and frequency of 55 mL, 2 s and 30 s (55/2/30), with bell shaped profile, 27.5 ml/s puff velocity, and hole vents blocked), accredited under ISO/TR 19478-2:2015^30^.The LM1 is a direct exposure system and does not perform smoke dilution unlike the smoking machine used by Rudd and colleagues. The LM4E Vaping Machine (Borgwaldt, Hamburg, Germany) (Fig. S6B) was used to vape *my*blu following the “CORESTA Reference Method n. 81” (CRM81) regimen (55 ml puff volume, drawn over 3 s, once every 30 s with square shaped profile, with a puff velocity of 18.3 ml/s), accredited into ISO 20,768:2018^31^. The standard exhaust time for both LM1 and LM4E was 0.7 s with a flow rate in the exposure chamber of 78.57 ml/s.

**Figure 6:**
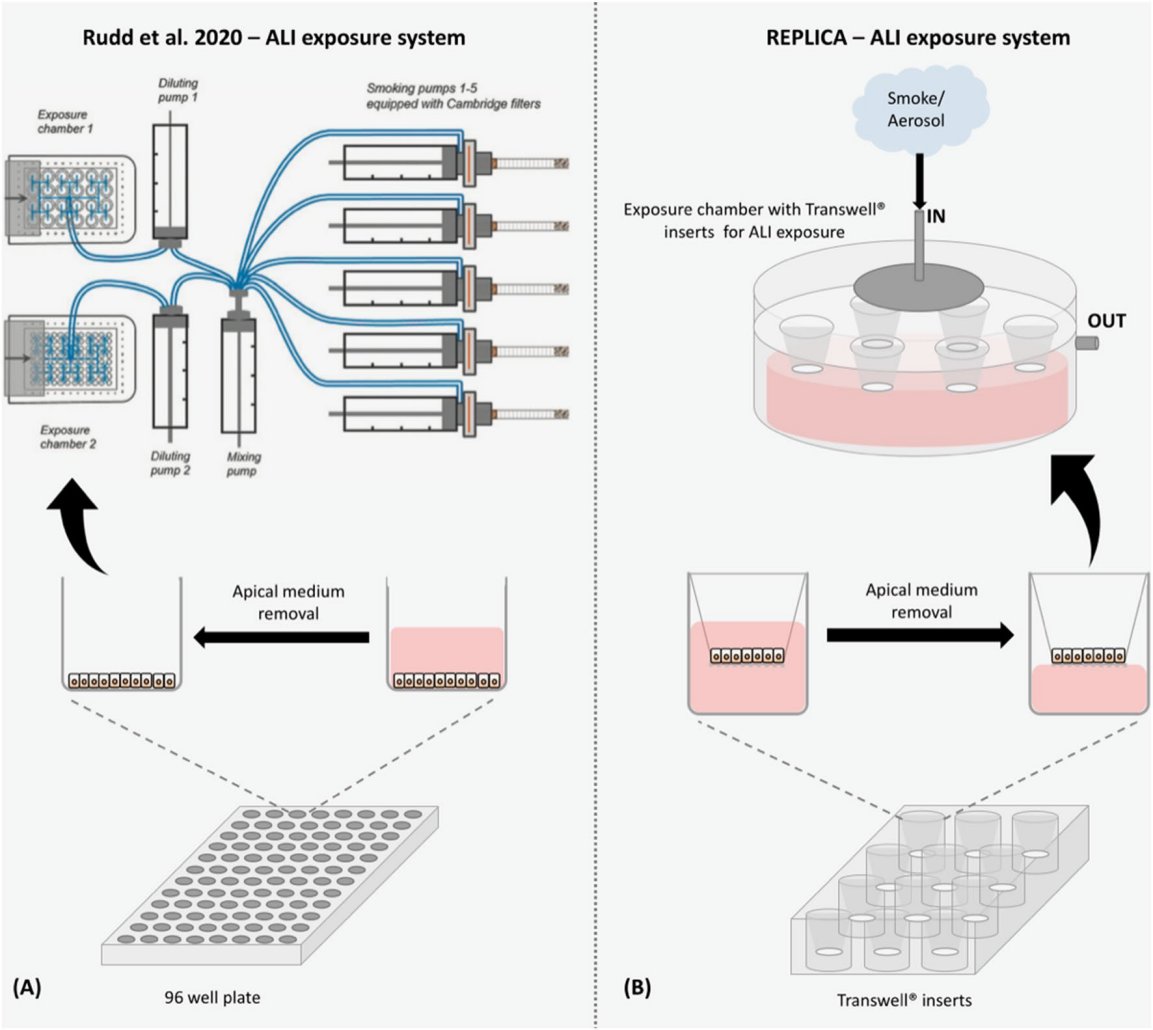
Air–liquid interface (ALI) exposure systems used by Rudd et al. (2020) and by this replication study in order to perform NRU assay. (**A**) Rudd et al. (2020) used the 96-well plate and they removed the culture medium from each well to expose BEAS-2B cells at the ALI. (**B**) In this study, the culture medium is removed from the upper part of the Transwell inserts and then placed in the exposure chambers on a plastic support that allows the cells to remain basally wet with medium and to be exposed to the smoke/vapor apically by the LM1/LM4 machines. The BAT exposure chamber allows a symmetrical aerosol distribution by the disc and ensures uniform cellular ALI exposure avoiding the accumulation of aerosol inside the system.

An air–liquid exposure system different from the original paper by Rudd et al. (2020) was used to expose BEAS-2B to perform NRU assay and V79 cells to perform IVMN assay. The BAT (British American Tobacco) aerosol exposure chambers (Fig. S7) were used to expose in vitro cells at the air–liquid interface (ALI) to combustible cigarette smoke and aerosols from e-cigarettes. In particular, cells cultured in Transwell inserts were deprived of the apical medium and placed in the exposure chambers containing the medium in the lower compartment, keeping wet the basal face of the cells, and then connected to the smoking or vaping machine to deliver undiluted whole smoke or whole aerosol to the apical face of cells (Fig. 6)^13^.

For the cytotoxicity evaluation of BEAS-2B, 1R6F cigarette smoke was delivered undiluted from 1 to 8 puffs (exposure time from 00:02 to 03:46 mm:ss). The *my*blu aerosol was delivered undiluted from 20 to 140 puffs (exposure time from 10:30 to 76:30 mm:ss), as reported by Rudd et al. (2020). Cytotoxicity evaluation was also performed to establish the EC_50_ of V79 cells exposed to 1R6F undiluted smoke prior to the IVMN assay. In that case, V79 cells were exposed to 1R6F smoke from 2 to 30 puffs (exposure time from 00:34to 15:30 mm:ss). Based on the EC50 previously calculated for the V79 cells on the results obtained in the NRU assay, we performed the 1R6F smoke exposure for the IVMN assay delivering from 1 to 4 puffs to the cells (exposure time from 00:02 to 01:38 mm:ss). The *my*blu aerosol was delivered undiluted from 20 to 100 puffs for the IVMN assay (exposure time from 10:30 to 54:30 mm:ss), as reported by Rudd et al. (2020). The experiments on cytotoxicity were replicated by the laboratory from Italy (CoEHAR, University of Catania, subsequently referred as LAB-A) and by the laboratory from Oman (Sultan Qaboos University, referred as LAB-B).

For the Ames assay, 1R6F cigarette smoke and the relative AIR control, or *my*blu aerosol and the relative AIR control, were delivered to the bacterial suspensions contained into their corresponding impingers at room temperature under protection from direct light. A puff with filtered ambient air was applied between smoke or aerosol puffs. The number of puffs is reported in Table S7 (supplementary material) for both the 1R6F exposure and for the *my*blu exposure. In particular, the impinger inlet for the 1R6F smoke exposure was connected to LM1 smoking machine (1R6F smoke) and to a LM4E channel equipped with a 44 mm Cambridge Filter Pad (CFP) (AIR interpuff) by means of a double one-way valve, whereas the impinger inlet for the *my*blu e-cigarette aerosol exposure was connected to two different LM4E channels, one of which equipped with e-cigarette and the other with a 44 mm CFP (AIR interpuff).

### Cell cultures

Normal bronchial epithelial cells (BEAS-2B / ATCC-CRL-9609) were cultured in collagen coated flasks using the bronchial epithelial growth medium supplemented with Lonza Bullet Kit (BEGM, Lonza CC-3170), as described by ATCC culture instructions. Hamster lung fibroblast cells (V79 / ICLC-AL99002) were cultured using Dulbecco's Modified Eagle Medium–high glucose (DMEM-hg, Thermo Fisher Scientific) with 10% FBS, 2 mM L-Glutamine, 50 U/mL penicillin, and 50 μg/mL streptomycin, as described by ICLC (Interlab Cell Line Collection; http://bioinformatics.hsanmartino.it/iclc/) instructions.

### Cytotoxicity evaluation: NRU assay

Cytotoxicity evaluation was performed using the BEAS-2B cells by using the NRU assay^8,32^. Moreover, cytotoxicity evaluation was performed with 1R6F whole smoke for the V79 cells, prior to genotoxicity evaluation, in order to establish the number of puffs to be used in the IVMN assay.

Prior to exposure, 300 μl of BEAS-2B cell suspension (BEGM supplemented with Lonza Bullet Kit and with 20 mM of HEPES buffer) was seeded in 24-well Transwell inserts at density of 150.000 cells/well and incubated for 20 ± 3 h. After incubation, the apical cell culture medium was removed, and the Transwell inserts were transferred into the corresponding exposure chamber filled with 25 ml of DMEM-hg supplemented with 50 U/mL penicillin and 50 μg/mL streptomycin in order to proceed to the smoke/vapor ALI exposure. After ALI exposure, each insert was transferred in a new 24-well plate filled with 500 μl and 300 μl of fresh BEGM (supplemented with Lonza Bullet Kit + 20 mM of HEPES buffer) respectively at the basal and apical compartments. Next, the cells were incubated for a recovery period of 65 ± 2 h^7^. The day before NRU assay, the NRU solution was prepared in BEGM medium at ratio 1:65 (0.05 g/L) plus HEPES buffer at 20 mM and placed in incubator at 37 °C 5% CO_2_. The day of NRU assay, the NRU solution was filtered prior to use. The culture medium was removed from the apical and basal compartments of each culture insert. The cells were washed twice with pre-warmed PBS, then incubated with neutral red solution (500 μl at the bottom and 300 μl at the top) for 3 h at 37 °C in 5% CO_2_ and a humidified atmosphere. After incubation, cells were washed twice with pre-warmed PBS to remove unincorporated dye. The incorporated solution was eluted from the cells by adding 330 μl of destain solution (50% ethanol, 49% distilled water, 1% glacial acetic acid v:v:v) to each insert and incubated for 10 min at 300 rpm on a plate shaker. Extracts were transferred to a 96-well plate in duplicate (100 μl per well) and optical density of neutral red extracts was read with a microplate spectrophotometer at 540 nm using a reference filter of 630 nm. Blank inserts (without cells) were used to assess how much neutral red solution stained the Transwell membranes and the mean of background values was subtracted from each measurement.

The same procedure was used for the cytotoxicity evaluation of V79 cells. In brief, 300 μl of V79 cell suspension (DMEM-hg supplemented with 10% FBS, 2 mM L-Glutamine, 50 U/mL penicillin, 50 μg/mL streptomycin, and 20 mM HEPES) was added in 24-well Transwell inserts at density of 100.000 cells/well and incubated for 24 h. The day of ALI exposure, the Transwell inserts (without apical cell culture medium) were transferred into the corresponding exposure chamber filled in the basal compartment with 25 ml of DMEM-hg supplemented with 50 U/mL penicillin and 50 μg/mL streptomycin. After ALI exposure, each insert was transferred in a new 24-well plate filled with 500 μl and 300 μl of fresh DMEM-hg (supplemented with 10% FBS, 2 mM L-Glutamine, 50 U/mL penicillin, 50 μg/mL streptomycin, and 20 mM HEPES) respectively at the basal and apical compartments. Next, the cells were incubated for a recovery period of 24 h. The NRU solution was prepared in DMEM-hg at ratio 1:65 (0.05 g/L) plus HEPES buffer at 20 mM. The next steps of NRU assay for V79 cells were the same as BEAS-2B and are described above. Three negative controls were performed for this assay: (i) cells were maintained in the incubator with both the basal and apical culture medium (INC), (ii) the cells were maintained in the incubator without the apical medium to reproduce the air–liquid interface exposure (ALI) and (iii) the cells were exposed to puffs of particulate-filtered laboratory air (AIR).

### Mutagenicity evaluation: Ames assay

The in vitro mutagenic effect of fresh 1R6F smoke and *myblu* aerosols was determined using Ames test^33^ as described by Rudd et al.^7^ with some modifications, and it was conducted only by LAB-A. The Ames screen was employed using only *S. typhimurium* TA98 and TA100 strains (Trinova Biochem GmbH) ± S9 treatment, which are the most sensitive to combustion products. The Ames assay was conducted in accordance with OECD (Organization for Economic Cooperation and Development) test guideline 471^9^.

Briefly, bacterial cultures of the TA98 and TA100 strains were prepared in 25 mL Nutrient Broth No.2 (OXOID) by inoculating one bacterium- followed by incubation overnight at 37 °C with shaking at 120 rpm. Then, bacterial suspensions were prepared by centrifugation of 25 mL cultures at 1800 g for 20 min at 4 °C, and the pellet was resuspended in 12 mL of Ca^2+^, Mg^2+^-free Dulbecco’s phosphate buffered saline (PBS). Next, 10 mL of the bacterial suspensions were placed in the corresponding impingers and exposed to test aerosols/smoke and filtered ambient air (negative control of exposure) as described above (exposed Bacterial Suspension or eBS). For each experiment, an aliquot of PBS with untreated PBS bacterial suspension (for the assay without S9 mix) and S9 mix with untreated PBS bacterial suspension (for the assay with S9 mix) as internal negative controls (Solvent control).

After each exposure, the bacterial suspensions were immediately used for Ames screening by following manufacturer’s protocol (Salmonella Mutagenicity Test Kit, MOLTOX®). Briefly, an aliquot of bacterial suspensions and relative reagents were added to sterile 15 mL test tubes as described in Table S8 (supplementary material). The solution was thoroughly mixed and then decanted on top of a minimal glucose agar plate, covered and set aside to solidify. When the top agar was solidified, the plates were inverted and placed in an incubator at 37 °C. After 48–72 h of incubation, the number of revertant colonies growing on the plates was counted manually.

Controlchem™ Mutagens were used as positive controls for both *S. typhimurium* stains TA98 and TA100 (see Table S9 of supplementary material). Each concentration of test aerosols or smoke and positive controls was tested in triplicate.

### Genotoxicity evaluation: IVMN assay

The IVMN assay was performed in concordance with OECD test guideline no. 487 ^10^ with and without S9 metabolic activation, and it was conducted only by LAB-A. Genotoxicity evaluation of whole fresh tobacco smoke and e-cigarette aerosol was performed using the hamster lung V79 cell line (Interlab Cell Line Collection (ICLC)-AL99002). The day before cell exposure, V79 cells were seeded in 24-well Transwell inserts (0.4 µm pore membrane) at density of 10 × 10^4^ with 200 µl of DMEM-hg supplemented with 10% of FBS. The inner wells of each 24-well plate were filled with 500 µl of DMEM-hg supplemented with 10% of FBS. The V79 cells were incubated for 24 h at 37 °C and 5% CO_2_. After 24 h of incubation, the apical medium was removed, and the inserts were transferred into the exposure chamber filled with 25 ml of DMEM-hg with the addition of HEPES buffer (20 mM final concentration) in the basal compartment. The exposure to 1R6F whole smoke and myblu e-cigarette whole aerosol is described in the previous section on “Smoke and vapour exposure". After the exposure, each insert was transferred in a new 24-well plate filled with 500 µl of DMEM-hg supplemented with HEPES buffer (20 mM). For the IVMN with S9, each insert containing exposed V79 cells, was filled with 300 µl of S9 mix at 10% in the apical compartment, and then incubated for 3 h at 37 °C. After incubation, the apical S9 medium was removed, the V79 cells were covered with DMEM-hg supplemented with HEPES buffer (20 mM) and incubated for 24 h to allow for at least one cell division cycle. For the IVMN without S9, inserts with exposed V79 cells were filled with DMEM-hg supplemented with HEPES buffer (20 mM) and incubated for 24 h at 37 °C and 5% CO_2_. Three negative controls were performed for both IVMN with and without S9 activation: (i) cells were maintained in the incubator with both the basal and apical culture medium (INC), (ii) the cells were maintained in the incubator without the apical medium to reproduce the air–liquid interface exposure (ALI) and (iii) the cells were exposed to puffs of particulate-filtered laboratory air (AIR). Positive controls, including cyclophosphamide A (CAS 6055–19-2) for the S9 fraction and mitomycin C (CAS 50–07-7) for the IVMN without S9, were used. All the tested conditions were assessed in triplicates.

After 24 h of recovery, the cells were detached and counted using the Muse® Cell Analyzer using the Muse® Count & Viability Kit (Luminex Corp.). The V79 cells were then seeded in 96-well plate (CellCarrier Ultra-96 Black, Optically Clear Bottom—PerkinElmer) at density of 10 × 10^3^ per well and incubated for 24 h. Next, the cells were fixed with 4% (PFA) (paraformaldehyde) for 20 min at room temperature. After fixation, the cells were washed once with PBS and then stained with DAPI (1 μg/mL). Micronuclei assessment was performed by using Harmony® High-Content Imaging and Analysis Software (PerkinElmer).

### Statistics

All raw data were collected and processed using Excel software (Microsoft, Redmond, WA, USA). R version 3.4.3 (2017-11-30) was used to assess reproducibility of NRU data between LAB-A and LAB-B. Reproducibility of NRU data obtained by LAB-A and LAB-B were evaluated by linear regression analysis of cell viability percentages (to AIR controls) between the two laboratories. Moreover, the mean differences and the limits of agreement (95% confidence interval) were calculated to assess the agreement between LAB-A and LAB-B and visualized by Bland–Altman plots. A 1-tailed sample T test was also performed to assess the mean differences between the two laboratories from zero (Table S10 and Fig. S5 of supplementary material).

For the cytotoxicity evaluation (NRU), data were expressed as percentage to the AIR control. The EC_50_ values for each exposure (1R6F and *my*blu) were calculated by fitting a sigmoidal dose–response curve with a variable slope to determine the best fit values for the 1R6F log EC_50_ of 7 parameter nonlinear regression model and for the *my*blu log EC_50_ of 7 parameter nonlinear regression model. Moreover, comparison of *my*blu results and AIR control was carried out by ANOVA followed by Dunnett’s post hoc multiple comparison test.

Data from Ames assay (mutagenicity evaluation) were reported as fold change relative to AIR control^3^, and calculated as follow:

$$\left( {{\text{revertant}}\;{\text{number}}\;{\text{relative}}\;{\text{to}}\;{\text{puff}}\;{\text{number}}} \right){-}\left( {{\text{mean}}\;{\text{of}}\;{\text{revertant}}\;{\text{number}}\;{\text{relative}}\;{\text{to}}\;{\text{AIR}}\;{\text{control}}} \right)/{\text{mean}}\;{\text{of}}\;{\text{revertant}}\;{\text{number}}\;{\text{relative}}\;{\text{to}}\;{\text{AIR}}\;{\text{control}}.$$

Linear regression analyses for each strain were performed to evaluate the mutagenic activity. Moreover, comparisons among 1R6F and respective controls and among *my*blu and respective controls were performed by using mixed-effect model or ANOVA followed by Tukey’s post hoc multiple comparison test.

Genotoxicity data were analyzed by linear regression of 1R6F or *my*blu dose–response slopes with comparison between slopes. Comparisons among the different dose of smoke or aerosol and respective controls were performed by ANOVA followed by Tukey’s (IVMN without S9 activation) or Dunnett’s (IVMN with S9 activation) post hoc multiple comparison tests.

All analyses were considered significant with a p value < 0.05. GraphPad Prism 8 software was used for data analysis and generation of graphs unless otherwise stated. Raw data were shared by Zenodo repository (https://doi.org/10.5281/zenodo.8335201).

## Additional Information

All experiments were performed in accordance with relevant guidelines and regulations. No animals or human tissue samples were used for the experiments. Cells from American Type Culture Collection (Manassas, VA, USA) were used for the experiments on cytotoxicity: Human normal bronchial epithelial cells (BEAS-2B / ATCC-CRL-9609). Hamster lung V79 cell line (ICLC-AL99002) from IRCCS Ospedale Policlinico San Martino, (Interlab Cell Line Collection—ICLC) were used for genotoxicity assessment. The pre-print version of this article is present on https://www.biorxiv.org/content/10.1101/2022.10.28.514205v2. This article is not published nor is under publication elsewhere.

### Supplementary Information

Supplementary Information.

## Data Availability

The datasets generated during the current study are available from the corresponding author on reasonable request.
