# Author Correction: Cytotoxicity, mutagenicity and genotoxicity of electronic cigarettes emission aerosols compared to cigarette smoke: the REPLICA project

**DOI:** 10.1038/s41598-024-53036-w

**Published:** 2024-05-01

**Authors:** Rosalia Emma, Virginia Fuochi, Alfio Distefano, Konstantinos Partsinevelos, Sonja Rust, Fahad Zadjali, Mohammed Al Tobi, Razan Zadjali, Zaina Alharthi, Roberta Pulvirenti, Pio Maria Furneri, Riccardo Polosa, Ang Sun, Massimo Caruso, Giovanni Li Volti, the Replica Project Group, Giovanni Li Volti, Massimo Caruso, Rosalia Emma, Antonio Giordano, Ang Sun, Vladislav Volarevic, Ronny Lesmana, Konstantinos Poulas, Alfio Distefano, Konstantinos Partsinevelos, Roberta Pulvirenti, Aurora Costa, Aleksandar Arsenijevic, Melisa I. Barliana, Konstantinos Mesiakaris, Najwa Albalushi, Chiara Giardina, Salvatore Furnari

**Affiliations:** 1 Department of Clinical and Experimental Medicine, University of Catania, Via S. Sofia, 97, 95123 Catania, Italy; 2 Center of Excellence for the Acceleration of Harm Reduction (CoEHAR), University of Catania, Via S. Sofia, 97, 95123 Catania, Italy; 3 Department of Biomedical and Biotechnological Sciences, University of Catania, Via S. Sofia, 97, 95123 Catania, Italy; 4 ECLAT Srl, Spin Off of the University of Catania, Via. S Sofia 89, 95123 Catania, Italy; 5 Department of Clinical Biochemistry, College of Medicine and Health Sciences, Sultan Qaboos University, P.C 123, P.O. Box 35, Khodh, Oman; 6 Department of Biology, College of Science and Technology, Sbarro Institute for Cancer Research and Molecular Medicine, Temple University, Philadelphia, USA; 7 Faculty of Medical Sciences, Center for Molecular Medicine and Stem Cell Research, University of Kragujevac, Kragujevac, Serbia; 8 Center of Excellence for Pharmaceutical Care Innovation, Universitas Padjadjaran, Jl. Raya Bandung Sumedang KM. 21, Jatinangor, 45363 Indonesia; 9 Department Biomedical Sciences, Faculty of Medicine, Universitas Padjadjaran, Jl. Raya Bandung Sumedang KM. 21, Jatinangor, 45363 Indonesia; 10 Institute for Research and Innovation, IRIS, Patras Science Park, Patras, Greece; 11 Laboratory of Molecular Biology and Immunology, Department of Pharmacy, University of Patras, Patras, Greece; 12 Department of Biological Pharmacy, Biotechnology Laboratory, Faculty of Pharmacy, Universitas Padjadjaran, Jl. Raya Bandung Sumedang KM. 21, Jatinangor, 45363 Indonesia

Correction to: *Scientific Reports* 10.1038/s41598-023-44626-1, published online 30 October 2023

The original version of this Article contained an error in Figure 6, where the pink colour representing the medium within the transwell inserts was not visible. The original Figure 6 and the accompanying legend appear below.

**Figure 6:**
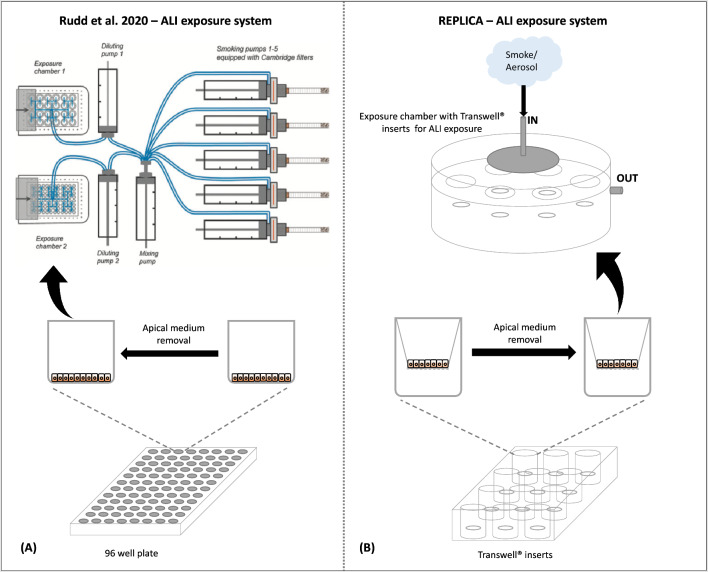
Air–liquid interface (ALI) exposure systems used by Rudd et al. (2020) and by this replication study in order to perform NRU assay. (**A**) Rudd et al. (2020) used the 96-well plate and they removed the culture medium from each well to expose BEAS-2B cells at the ALI. (**B**) In this study, the culture medium is removed from the upper part of the Transwell inserts and then placed in the exposure chambers on a plastic support that allows the cells to remain basally wet with medium and to be exposed to the smoke/vapor apically by the LM1/LM4 machines. The BAT exposure chamber allows a symmetrical aerosol distribution by the disc and ensures uniform cellular ALI exposure avoiding the accumulation of aerosol inside the system.

The original Article has been corrected.

