# Supplementary material for: Cytotoxicity, mutagenicity and genotoxicity of electronic cigarettes emission aerosols compared to cigarette smoke: the REPLICA project
Source: Sci Rep. 2023 Oct 30;13:17859. doi: 10.1038/s41598-023-44626-1 (PMC10616076; doi:10.1038/s41598-023-44626-1)
Supplement: Supplementary file 1 — Supplementary Information. [file 41598_2023_44626_MOESM1_ESM.docx]

**Supplementary material**

**Results**

**Evaluation of BEAS-2B morphology after exposure to 1R6F smoke and myblu vapor**

**Figure S1**. Morphological evaluation of BEAS-2B cells after exposure to 1R6F cigarette smoke.

**Figure S2.** Morphological evaluation of BEAS-2B cells after exposure to myblu vapor.

**Mutagenicity effect of 1R6F cigarette smoke and *my*blu aerosol**

**Table S1.** Linear regression analysis of mutagenic activity in Salmonella tiphimurium (TA98 and TA100) with S9 metabolic activation.

| **Strain** | **Product** | **Slope ± SE** | **95% Confidence Interval** | **Is Slope Significantly Non-Zero? Yes/No (P Value)** | **Are The Slopes Equal? Yes/No (P Value)** |
| --- | --- | --- | --- | --- | --- |
| **TA 98** | 1R6F | 0.0596 ± 0.0099 | 0.0382 to 0.0809 | YES (p<0.0001) | NO (p< 0.0001) |
|  | myblu | 0.0004 ± 0.0008 | -0.00139 to 0.00219 | NO (p= 0.637) |  |
| **TA 100** | 1R6F | 0.01407 ± 0.004 | 0.00638 to 0.0218 | YES (p= 0.0017) | NO (p= 0.0002) |
|  | myblu | 0.00109 ± 0.0003 | 0.0004 to 0.001 | YES (p= 0.0045) |  |

**Table S2.** Linear regression analysis of mutagenic activity in Salmonella tiphimurium (TA98 and TA100) without S9 metabolic activation.

| **Strain** | **Product** | **Slope ± SE** | **95% Confidence Interval** | **Is Slope Significantly Non-Zero? Yes/No (P Value)** | **Are The Slopes Equal? Yes/No (P Value)** |
| --- | --- | --- | --- | --- | --- |
| **TA 98** | 1R6F | 0.07815 ± 0.01045 | 0.0556 to 0.1007 | YES (p<0.0001) | NO (p< 0.0001) |
|  | myblu | -0.0001 ± 0.001 | -0.003 to 0.002 | NO (p= 0.91) |  |
| **TA 100** | 1R6F | 1.102 ± 0.2120 | 0.6440 to 1.560 | YES (p= 0.0002) | NO (p< 0.0001) |
|  | myblu | 0.00095 ± 0.0002 | 0.0005 to 0.0014 | YES (p= 0.0005) |  |

**Genotoxicity effect of 1R6F cigarette smoke and myblu aerosol**

**Figure S3** Cytotoxicity evaluation of V79 cells after exposure to undiluted 1R6F cigarette smoke*.* The calculated EC50 value was 3.149 puffs (Log EC50= 0.498). Evaluation of cytotoxicity for IVM assay according to OECD n.487 guideline.

**Table S3**. IVM assay with S9 metabolic activation of 1R6F whole smoke

| **Conditions** | **RICC** | **Population doubling** | **RPD** |
| --- | --- | --- | --- |
| AIR | 90.702 % | 1.081 | 96.591 % |
| 1R6F 1puff | 59.542 % | 0.767 | 68.553 % |
| 1R6F 2puff | -32.370 % | -0.711 | -63.531 % |
| 1R6F 3 puff | -35.401 % | -0.864 | -77.212 % |
| 1R6F 4 puff | -42.164 % | -1.062 | -94.968 % |
| Cyclophosphamide A | 57.076 % | 0.746 | 66.641 % |
| RICC: Relative Increase in Cell Counts; RPD: Relative Population Doubling. | | | |

**Table S4.** IVM assay with S9 metabolic activation of myblu vapor

| **Conditions** | **RICC** | **Population doubling** | **RPD** |
| --- | --- | --- | --- |
| AIR | 65.009 % | 2.069 | 81.282 % |
| myblu 20 puff | 55.975 % | 1.898 | 74.557 % |
| myblu 40 puff | 41.545 % | 1.528 | 60.020 % |
| myblu 60 puff | 67.584 % | 2.108 | 82.825 % |
| myblu 80 puff | 76.681 % | 2.248 | 88.334 % |
| myblu 100 puff | 68.869 % | 2.123 | 83.420 % |
| Cyclophosphamide A | 72.402 % | 2.162 | 84.933 % |
| RICC: Relative Increase in Cell Counts; RPD: Relative Population Doubling. | | | |

**Figure S4**. Cytotoxity evaluation of S9 mix on V79 cells. ***p< 0.001; ***p< 0.0001

**Table S5.** IVM assay without S9 metabolic activation of 1R6F whole smoke

| **Conditions** | **RICC** | **Population doubling** | **RPD** |
| --- | --- | --- | --- |
| AIR | 80.569 % | 0.888 | 89.896 % |
| 1R6F 1puff | 52.133 % | 0.605 | 61.251 % |
| 1R6F 2puff | -30.806 % | -0.576 | -58.342 % |
| 1R6F 3 puff | -5.924 % | -0.104 | -10.491 % |
| 1R6F 4 puff | -10.664 % | -0.197 | -19.931 % |
| Mitomycin C | 85.308 % | 0.913 | 92.516 % |
| RICC: Relative Increase in Cell Counts; RPD: Relative Population Doubling. | | | |

**Table S6.** IVM assay without S9 metabolic activation of myblu vapor

| **Conditions** | **RICC** | **Population Doubling** | **RPD** |
| --- | --- | --- | --- |
| AIR | 106.667 % | 2.804 | 103.103 % |
| myblu 20 puff | 106.667 % | 2.803 | 103.075 % |
| myblu 40 puff | 112.889 % | 2.878 | 105.824 % |
| myblu 60 puff | 100.741 % | 2.638 | 97.004 % |
| myblu 80 puff | 64.593 % | 2.212 | 81.347 % |
| myblu 100 puff | 91.111 % | 2.615 | 96.155 % |
| Mitomycin C | 108.000 % | 2.819 | 103.667 % |
| RICC: Relative Increase in Cell Counts; RPD: Relative Population Doubling. | | | |

**Methods**

**Smoke and vapour exposure**

**Table S7.** Exposure conditions for the bubbling of bacterial suspension (AMES assay)

| **Conditions** | **Puff number (LM1)** | **AIR interpuff number (LM4E)** | **Conditions** | **Puff number (LM4E port 1)** | **AIR interpuff number (LM4E port 2)** |
| --- | --- | --- | --- | --- | --- |
| **1R6F (HCI regime)** | | | ***myb*lu e-cig (CRM81 regime)** | | |
| 1 cig | 9 | 9 | 60 puffs | 60 | 60 |
| 2 cigs | 18 | 18 | 120 puffs | 120 | 120 |
| 3 cigs | 27 | 27 | 180 puffs | 180 | 180 |
| 4 cigs | 36 | 36 | 240 puffs | 240 | 240 |
| 5 cigs | 45 | 45 | 300 puffs | 300 | 300 |
| **AIR Control (1R6F) – HCI** | | | **AIR Control (*myb*lu e-cig) – CRM81** | | |
| 1 cig | 9 | / | 60 puffs | 60 | / |
| 2 cigs | 18 | / | 120 puffs | 120 | / |
| 3 cigs | 27 | / | 180 puffs | 180 | / |
| 4 cigs | 36 | / | 240 puffs | 240 | / |
| 5 cigs | 45 | / | 300 puffs | 300 | / |

**Mutagenicity evaluation: AMES assay**

**Table S8.** Schematic representation of the assay. The example is intended for one bacterial strain only and for both S9- and S9+ mix.

| **Assay mix** | **S9-** | | | | **S9+** | | | |
| --- | --- | --- | --- | --- | --- | --- | --- | --- |
|  | **eBS** | **Air Control** | **Solvent control** | **Controlchem** | **eBS** | **Air Control** | **Solvent control** | **Controlchem** |
| **mL of melted agar for tube** | 2 ml | | | | | | | |
| **Components to add into melted agar** | 200 μL of eBS | 200 μL of eBS air control | 100μL of PBS + 100 μL of the untreated PBS bacterial suspension | 100μL of controlchem solution + 100μL of untreated PBS bacterial suspension | 500μL S9 mix and 50μL of eBS | 500μL S9 mix and 50μL of eBS air control | 500μL S9 mix +50μL of the untreated eBS bacterial suspension | 500μL S9 mix + 100μL of controlchem solution + 50μL of the untreated PBS bacterial suspension |
|  | ↓ | | | | | | | |
|  | Minimal Glucose Agar plates | | | | | | | |
| eBS: exposed Bacterial Suspension | | | | | | | | |

**Table S9.** Controlchem™ Mutagens and Related Solvents.

| **Mutagen** | **Amount (μg)** | **Strain** | **Solvent** |
| --- | --- | --- | --- |
| Sodium Azide (S9- chemcontrol for TA100) | 15 | TA100 | Water |
| Daunomycin (S9- chemcontrol for TA98) | 60 | TA98 | Water |
| 2-Aminoanthracene (S9+ activation control) | 100 | Both | DMSO |

**Data reproducibility of NRU results between LAB-A and LAB-B**

Linear regression analyses of NRU cell viability results showed good reproducibility for both 1R6F smoke exposure and *my*blu™ vapor exposure between the two laboratories (Table S1). The mean of the cell viability difference for 1R6F was not different from zero (p= 0.058). Instead, the mean of the cell viability difference for *my*blu was significant different from zero (p= 0.015) despite being lower than the 1R6F mean difference (Table S1).

**Table S10**. Results of linear regression and one sample analyses between LAB-A and LAB-B.

|  | Regression analysis LAB-A − LAB-B  R value (p value) | Mean of the difference being different from zero? | |
| --- | --- | --- | --- |
|  |  | Mean of the Difference (95% CI) | YES/NO (p value*) |
| 1R6F | 0.953 (p= 0.004) | -11.9 (-31.7/7.9) | NO (p= 0.058) |
| *my*blu™ | 0.870 (p= 0.002) | -6.63 (-16.81/3.55) | YES (p= 0.015) |
| CI: confidence interval; *p value was calculated using the one sample T test. | | | |

The agreements between LAB-A and LAB-B cell viability results were evaluated by Bland-Altman plots, which showed the mean of two measurements from two laboratories against the single differences between laboratories results (Figure S1). All the differences between the two laboratories data were inside the limits of agreements.

**Figure S5**. Agreement assessment for NRU cell viability results after exposure to 1R6F (**A**) and myblu (**B**) between LAB-A and LAB-B. Bland Altman plots represent the difference between the measurements obtained by LAB-A and LAB-B (y-axis) with respect to the mean difference (x-axis). The dashed line in the middle of plot shows the average difference in cell viability between the two laboratories. The upper and lower dashed lines show the limits of agreement (95% confidence interval) between the results obtained from the two laboratories. No results from both 1R6F exposure

and myblu™ exposure had the cell viability (% of AIR Control) difference outside the 95% confidence interval.

**Figure S6**. LM1 smoking machine (A) and LM4E vaping machine (B)^1^

Figure S7. Perspex aerosol exposure chamber^1^

**REFERENCES**

1	Caruso, M. *et al.* Electronic nicotine delivery systems exhibit reduced bronchial epithelial cells toxicity compared to cigarette: the Replica Project. *Scientific Reports* **11**, 24182, doi:10.1038/s41598-021-03310-y (2021).
